# LJCD-Net: Cross-Domain Jamming Generalization Diagnostic Network Based on Deep Adversarial Transfer

**DOI:** 10.3390/s24113266

**Published:** 2024-05-21

**Authors:** Zhichao Zhang, Zhongliang Deng, Jingrong Liu, Zhenke Ding, Bingxun Liu

**Affiliations:** School of Electronic Engineering, Beijing University of Posts and Telecommunications, Beijing 100876, China; dengzhl@bupt.edu.cn (Z.D.); dingzk@bupt.edu.cn (Z.D.);

**Keywords:** jamming diagnosis, GNSS, 5G networks, deep domain generalization, pseudo-labeling, adversarial training

## Abstract

Global Navigation Satellite Systems (GNSS) offer comprehensive position, navigation, and timing (PNT) estimates worldwide. Given the growing demand for reliable location awareness in both indoor and outdoor contexts, the advent of fifth-generation mobile communication technology (5G) has enabled expansive coverage and precise positioning services. However, the power received by the signal of interest (SOI) at terminals is notably low. This can lead to significant jamming, whether intentional or unintentional, which can adversely affect positioning receivers. The diagnosis of jamming types, such as classification, assists receivers in spectrum sensing and choosing effective mitigation strategies. Traditional jamming diagnosis methodologies predominantly depend on the expertise of classification experts, often demonstrating a lack of adaptability for diverse tasks. Recently, researchers have begun utilizing convolutional neural networks to re-conceptualize a jamming diagnosis as an image classification issue, thereby augmenting recognition performance. However, in real-world scenarios, the assumptions of independent and homogeneous distributions are frequently violated. This discrepancy between the source and target distributions frequently leads to subpar model performance on the test set or an inability to procure usable evaluation samples during training. In this paper, we introduce LJCD-Net, a deep adversarial migration-based cross-domain jamming generalization diagnostic network. LJCD-Net capitalizes on a fully labeled source domain and multiple unlabeled auxiliary domains to generate shared feature representations with generalization capabilities. Initially, our paper proposes an uncertainty-guided auxiliary domain labeling weighting strategy, which estimates the multi-domain sample uncertainty to re-weight the classification loss and specify the gradient optimization direction. Subsequently, from a probabilistic distribution standpoint, the spatial constraint imposed on the cross-domain global jamming time-frequency feature distribution facilitates the optimization of collaborative objectives. These objectives include minimizing both the source domain classification loss and auxiliary domain classification loss, as well as optimizing the inter-domain marginal probability and conditional probability distribution. Experimental results demonstrate that LJCD-Net enhances the recognition accuracy and confidence compared to five other diagnostic methods.

## 1. Introduction

The Global Navigation Satellite System (GNSS) provides a wide-area, all-weather, real-time positioning and timing (PNT) service that estimates the position, navigation, and timing worldwide [1]. Currently, with the ongoing development of implicit application requirements, particularly for intelligent spatiotemporal networks, petrochemicals, and other multi-category application scenarios and service objects, there is an urgent need to establish a unified precise positioning and timing service system for the precise navigation and control of various types of less-manned and unmanned equipment. The demand for positioning continues to deepen from regional high precision to wide area high precision and high credibility [2].

**Figure 1:**
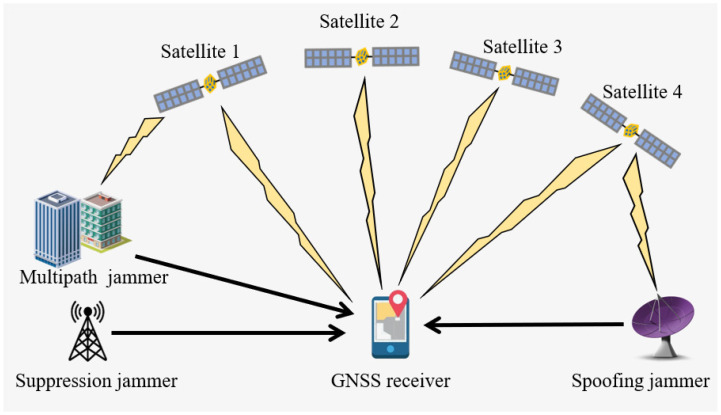
The performance of a localization receiver is influenced by jamming during the processing of Global Navigation Satellite System (GNSS) data.

Regardless of whether positioning is conducted via GNSS or a 5G communication navigation fusion signal [3], the terminal-side signal of interest (SOI) receives extremely low power, and intentional and unintentional jamming where the frequency bandwidth overlaps or is in close proximity to the SOI bandwidth and can have a destructive impact on the acquisition, tracking, and localization phases of the positioning receiver [4]. As illustrated in Figure 1, the two primary types of human-induced jamming are suppressive and deceptive. The former type possesses high power, preventing the receiver from locating by obstructing its ability to lock onto the SOI. The latter, by transmitting SOI in a specified format and amplifying the signal to an optimal power level, manipulates the receiver to lock on to the wrong signal, generating a false position solution. Typically, the spoofers are located in the vicinity of the localization receiver [5].

The objective of jamming detection and identification is to provide alerts and identify the type of jamming when a positioning receiver encounters disruptions. This process holds significant importance for enhancing the precision and dependability of a SOI in intricate positioning scenarios. However, the conventional methods of jamming recognition are constrained by empirical feature-based classification techniques that exhibit sensitivity to feature selection. Furthermore, these methods often lack adaptability across diverse tasks. Given that the core parameters of various jamming types vary, and their impacts on the signal’s time-frequency spectrum, power spectrum, or correlation peak diagram post-capture differ [6], it becomes imperative to develop specific parameter estimation and identification algorithms tailored for each type of jamming. Adaptive frequency-domain or time-domain filtering methods are commonly used for continuous wave (CW) jamming mitigation. Both frequency domain finite impulse response (FIR) and infinite impulse response (IIR) filtering techniques can effectively reduce jamming at specific frequencies. However, these methods necessitate prior knowledge of the jamming signal’s structure [6,7]. Ref. [8] examines the model of an array antenna’s reception signal, utilizing the correlation of the steering vector to identify and mitigate various forms of spoofing jamming. Most existing jamming diagnosis methods rely on manually selected feature parameters based on the distribution law of specific type jamming signals. Consequently, these algorithm models are limited to a single applicable scenario, leading to a substantial design workload and a lack of universality.

In recent years, the deepening of machine learning theory research has led to a surge in interest in deep learning, a method renowned for its robust information representation capabilities [9]. This approach has been successfully employed in various fields such as anomaly detection, image classification, and semantic segmentation. Ref. [10] proposed a new method for jamming identification using a residual network constructed in the time domain. The method converts the time domain signals into one-dimensional data as inputs to the network, but the amount of data is small and the network generalization ability is poor. However, the assumption of independent and identically distributed signals may frequently be violated in industrial application scenarios [11,12]. This is exemplified by the irregular relative motion between the interfering source and the localization receiver, which alters both the frequency and magnitude of jamming features. Furthermore, the integration of multipath factors [13] results in a shift in the relative positions of sample features on the spectrogram as the number of reflections within the same time frame increases. This leads to a decrease or even failure in the diagnostic performance of the original trained model when dealing with jamming samples exhibiting unknown distribution changes. In essence, training data with fully labeled source domain and unlabeled test data from the target domain share similar but distinct distributions. The inherent differences between the source and target domains can induce domain drift under varying working conditions, resulting in a reduction in model performance when applied for inference in the target domain after training in the source domain [14,15,16].

Domain adaptation (DA) seeks to address the issue of disparate source and target distributions [17]. DA assumes that the target data are available during the training process. However, in practical scenarios, it may be challenging to secure evaluation samples during this phase. Furthermore, in large-scale cross-modal and variable power jamming contexts, a single source domain transfer learning model tends to garner limited information. This leads to a marked deficiency in diagnostic accuracy. Merging cross-source domain data indiscriminately can disrupt the source distribution. Weakening the model’s ability to acquire precise knowledge, this compromises both the diagnostic efficacy of the model and its overall stability.

Domain generalization (DG) has garnered increased attention as a means to mitigate reliance on target domain data, offering the potential to extend trained models across multiple sources to unseen targets [18]. Using local Fisher discriminant analysis, multiple source domains are represented as points on the Grassmann manifold, and their average subspace is built to uncover generic diagnostic knowledge [19]. Current research in multisource domain generalization predominantly spans mechanical, medical, remote sensing, and other fields. To our understanding, this represents the inaugural work on deep adversarial domain generalization within the realm of jamming diagnosis.

We attempted to collect enough jamming information data in the laboratory for jamming diagnosis in real localization scenarios. Although DG methods show great potential for jamming diagnosis in unknown scenarios, existing methods focus on learning a large amount of shared knowledge with fully labeled source domain data during the training phase [20]. In practice, the number of unlabeled samples is much larger than labeled samples. Existing studies [21,22,23] have shown that the diagnostic performance of DG slips when the number of complete labeled sources used for training is limited.

**Figure 2:**
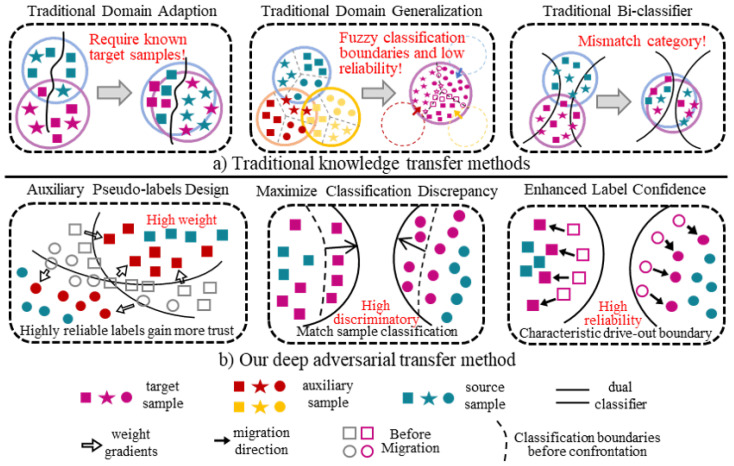
Illustration of (**a**) traditional knowledge migration method and (**b**) our deep adversarial migration method.

To address the above challenges, in this paper, we design a deep adversarial migration-based cross-domain jamming generalization diagnostic network (LJCD-Net), which aims to diagnose the target jamming using the generalized knowledge learned from a fully labeled source domain and multiple unlabeled domains (auxiliary domains). Figure 2 provides a comparative analysis of the prevalent transfer learning methods in current image processing fields, juxtaposing them with the approach proposed in this study. In contrast to conventional DA and DG methodologies, this study introduces a novel pseudo-label weighting strategy. This approach offers significant labeling guidance for unsupervised auxiliary samples without necessitating target labeling data. By optimizing the implicitly distributed adversarial learning model, the dual classifier is compelled to produce more accurate predictions. Consequently, this reduces the prevalence of fuzzy samples and propels features away from classification boundaries, thereby achieving highly discriminative and dependable jamming diagnosis.

LJCD-Net is executed in two distinct phases: auxiliary domain labeling design based on uncertainty bootstrapping and dual classifier adversarial learning based on implicit distribution alignment. In the initial stage, the short-time Fourier transform (STFT) is used to obtain the time-frequency local distribution of the jamming signal. The global attention mechanism residual module is introduced to amplify the global interaction representation, which reduces network degradation while focusing model training on the global spatial features and high-response channels of the time-frequency map. By estimating the uncertainty-weighted classification loss of pseudo-labels after knowledge aggregation, the classification loss of source domain samples is optimized while giving high-reliability labels to auxiliary domain samples. The second stage consists of two modules, domain alignment and category alignment. The domain discriminator distinguishes whether the samples belong to the auxiliary domain or the source domain, in order to maximize the confusion of different domain edge distributions to complete the domain alignment. The two interfering classifiers in the category alignment module design opposite loss gradient directions in their respective sample sets based on the quality of the decisions made in different samples, and the feature generator tries to reconcile the contradiction between the two interfering classifiers, and extracts shared domain-invariant features through the anti-training losses in the classifier stopping update phase. Meanwhile, the category adversarial metrics factor is designed based on the prediction results to improve the confidence of the classification discriminative ability, with the goal of obtaining the desired classification boundaries in the testing phase. Specifically, the contributions generated by this paper are summarized below.

- In order to solve the problem of data distribution gap in cross-domain jamming sensing scenarios of the model, a cross-domain jamming generalization diagnosis method based on deep adversarial transfer is proposed. It utilizes a fully labeled source domain and multiple unlabeled auxiliary domains to generate shared feature representations with generalization capabilities that do not rely on interfering samples on the target domain;
- Aiming at the problem of a lack of supervision of auxiliary domain samples and unreliable pseudo-label labeling methods in the generalization model, an auxiliary domain label weighting strategy based on uncertainty guidance is proposed. The estimation of the uncertainty of unsupervised samples is used to quantify their reliability weight to alleviate the negative impact caused by low-confidence pseudo-labels;
- A dual-classifier adversarial learning method based on implicit distribution alignment is proposed to solve the problems of poor discriminability and low confidence in model classification. This method spatially constrains the time-frequency feature distribution of cross-domain global jamming from a probability distribution perspective. The construction of a domain-level discriminative and class-confrontational implicit alignment module minimizes source domain classification loss, auxiliary domain classification loss, inter-domain edge probability, and conditional probability distribution, effectively reducing domain disagreements between different jamming modes.

The rest of the paper is organized as follows: Section 2 introduces a typical jamming signal model. Section 3 describes the proposed cross-domain jamming generalized diagnostic model in detail, including the generation of jamming time-frequency images, the design of pseudo-label weighting of samples in the auxiliary domain, and the implementation of implicit adversarial learning for dual classifiers. Section 4 describes in detail the dataset setup method. Section 5 evaluates the diagnostic performance of LJCD-Net in AM, FM, chirp, pulse, narrowband, and spoofing jamming under different conditions and experimentally analyzes the proposed method. Finally, the paper is summarized in Section 6.

## 2. Signal Models

After passing through the antenna, the SOI is converted down to the intermediate frequency at the radio frequency front end (RF). The instantaneous noise-free sampling signal at the *n*th moment is modeled as:

(1) $$S_{I}\left[n\right]=\sum_{k=1}^{K}A_{k}d_{k}\left(nT-\tau_{k}\right)c_{k}\left(nT-\tau_{k}\right)\times \cos\left(2\pi \left(f_{I}+f_{d_{k}}\right)nT+\phi_{k}\right)$$

In the formula, $T=1/f_{s}$ is the sampling period, $f_{S}$ is the sampling frequency, $f_{I}$ represents the intermediate frequency, *k* represents the number of satellites received, $A_{k}$ and $\tau_{k}$ represent the amplitude and delay of the *k*th SOI, respectively, $c_{k}\left(\cdot \right)$ and $d_{k}\left(\cdot \right)$ represents the pseudo-random spreading code and the navigation message bit stream, respectively, and $f_{d_{k}}$ and $\phi_{k}$ represent the Doppler frequency shift and phase deviation of the *k*th signal, respectively.

After RF, we consider the baseband signal, i.e., $f_{I}=0$. The instantaneous noise-free sampled signal at the *n*th moment is modeled as:

(2) $$y\left[n\right]=\sum_{k=1}^{K}A_{k}h_{k}\left[n\right]d_{k}\left(nT-\tau_{k}\right)c_{k}\left(nT-\tau_{k}\right)\times \cos\left(2\pi f_{d_{k}}nT+\phi_{k}\right)+z\left[n\right]+v\left[n\right]$$

where $h_{k}\left[n\right]$ is the channel parameter of the *k*th link, z[n] is the suppressed or spoofed jamming component, v[n] is an independent identically distributed Gaussian noise obeying a mean of 0 and a variance of $\sigma^{2}=N_{0}f_{s}/2$, and $N_{0}$ is the one-sided power spectral density of the noise. Furthermore, let p[n] and q[n] denote the channel coefficient vectors for suppressed and spoofed links, respectively.

This article explores research on five typical suppressive jammings. However, the influence of fading channels, Doppler frequency offset, and phase shift deviation on a SOI has been largely overlooked by scholars. Beyond a SOI, positioning receivers are also influenced by other environmental factors in the test environment, such as spoofing and obstruction from proximate fading environments like urban canyons and mountains. We have broadened the scope of jamming diagnosis to include not only typical suppressive jamming but also the quantity of spoofing and SOI. The latter serves as a measure of the complexity of the positioning environment; an increased number of obstacles results in fewer available signals. This paper proposes a more realistic signal model, incorporating Doppler, phase shift, and channel fading into the jamming diagnosis network. z[n] represents any of the following disturbances:

(1) AM Jammer: continuous Wave (CW) jamming encompasses both mono and poly AM signals. This typically pertains to signals with a bandwidth of no more than 10 KHz, expressed as:
  (3) $$z_{am}\left[n\right]=Pp\left[n\right]⊗\sum_{k=1}^{M_{a}}R_{p_{k}}\exp\left(j\left(2\pi f_{p_{k}}nT+\theta_{p_{k}}\right)\right)$$
  where $M_{a}$ is the number of AM polytones, *P* is the jamming gain coefficient calculated from the jamming-to-SOI power ratio (JSR), ⊗ is the convolution operator due to multipath fading in the interfering link, and $R_{p_{k}}$, $f_{p_{k}}$, and $\theta_{p_{k}}$ denote the amplitude, frequency, and phase corresponding to the *k*th AM jamming component, respectively.
(2) FM Jammer: The carrier frequency of the jamming is a time-dependent variable, denoted as:
  (4) $$z_{fm}\left[n\right]=Pp\left[n\right]⊗\sum_{k=1}^{M_{f}}a\cdot \exp\left(j\left(2\pi f_{m_{k}}nT+\beta_{k}\sin\left(2\pi f_{m_{k}}nT\right)\right)\right)$$
  Among them, $M_{f}$ is the number of multi-tone carriers in FM jamming, $f_{m_{k}}$ is the frequency of the *k*th jamming, and $\beta_{k}$ is the modulation index of the *k*th carrier.
(3) Chirp Jammer: The instantaneous intermediate frequency oscillates across a frequency band in a brief period, subsequently initiating another cycle of change. Two prevalent forms of chirp jamming are identified: linear chirp and sine wave chirp.
  (5) $$z_{cp}\left[n\right]=Pp\left[n\right]⊗\exp\left(j\left(2\pi f_{p}nT+\pi a\frac{f_{\max}-f_{\min}}{T_{swp}}\theta_{c}\right)\right)$$
  where $f_{p}$ is the initial sweep frequency, $f_{\min}$ and $f_{\max}$ are the minimum and maximum sweep frequency points, $T_{swp}$ is the time taken from $f_{\min}$ to $f_{\max}$, *a* is a random number with the value of 1 or −1 for determining the sweep direction, and $\theta_{c}$ is the initial phase of the chirp jamming.
(4) Narrowband (NB) noise Jammer: The power in the in-band spectrum significantly surpasses that of the SOI. Generally, the jamming bandwidth tends to be less than the primary lobe bandwidth of the SOI. The spectrum is articulated as follows:
  (6) $$\Omega \left(f\right)=\left\{\begin{array}{cc} \mu , & \left|f-f_{0}\right|<B/2\\ 0, & \mathrm{otherwise} \end{array}\right.$$
  where μ, $f_{0}$, and *B* are the amplitude, center frequency, and bandwidth of NB jamming, respectively. Filter the stationary random process to obtain the time series representation of narrow-band jamming ω[n], with $z_{nb}\left[n\right]=Pp\left[n\right]⊗\omega \left[n\right]$.
(5) Pulse Jammer: Multiple “duty cycles” are consistently transmitted within a repetitive temporal framework, characterized by their brief duration, expansive spectrum bandwidth, and potent burst power. This is represented as a sequence of Gaussian-shaped pulse pairs.
  (7) $$z_{pl}\left[n\right]=Pp\left[n\right]⊗\sum_{k=1}^{M_{p}}\alpha_{k}u\left(nT-\eta_{k}\right)\exp\left(j2\pi f_{k}\left(nT-\eta_{k}\right)\right)$$
  where u(·) represents the pulse waveform, $M_{p}$ represents the number of pulses in the duration time, and $\alpha_{k}$, $f_{k}$, and $\eta_{k}$ denote the amplitude, frequency, and time delay of the *k*th pulse, respectively.
(6) Spoofing signals: A spectrum jamming that matches the real SOI, usually with the same PRN pseudocode sequence as the SOI, but with false navigation message bits stored. Due to spatial heterogeneity, spoofing jamming and SOI often experience different delays and Doppler frequency offsets. The expression is as follows:
  (8) $$z_{sp}\left[n\right]=\sum_{k=1}^{K}R_{k}q_{k}\left[n\right]⊗{\hat{d}}_{k}\left(nT-\delta_{k}\right)c_{k}\left(nT-\delta_{k}\right)\times \cos\left(2\pi f_{kp}nT+\psi_{k}\right)$$
  where $R_{k}$ is the gain factor determined by the spoofing-to-noise power ratio, $q_{k}\left[n\right]$ is the channel coefficient vector corresponding to the *k*th PRN code transmitted by the spoofing link, ${\hat{d}}_{k}$ denotes the false navigation message information sent, and $\delta_{k}$, $f_{kp}$, and $\psi_{k}$ are the delay, Doppler shift, and phase deviation, respectively, for spoofing to interfere with the *k*th PRN code sequence.

In the absence of jamming, the true signal received contains only the true SOI and white thermal noise. Since the SOI entering the antenna is much weaker than the thermal noise before the spreading correlation, the spectrum of the mixed signal is then determined by the thermal noise.

## 3. Proposed Cross-Domain Jamming Generalization Diagnosis Network

**Figure 3:**
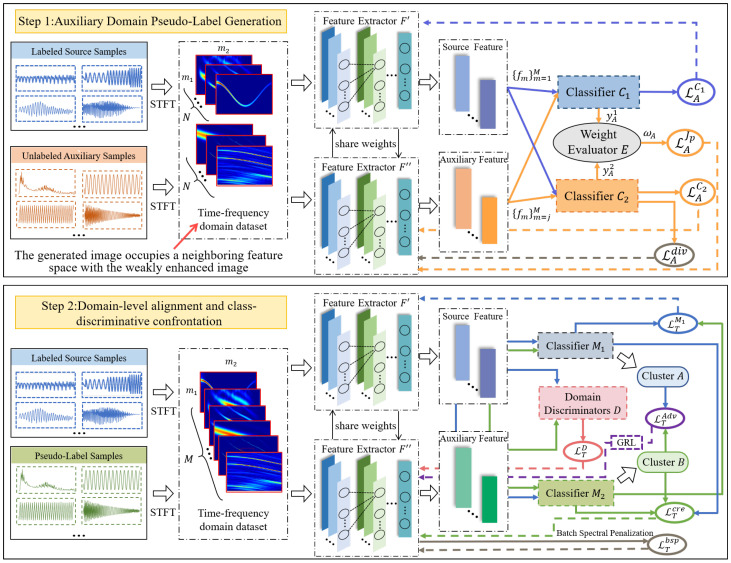
Our two-stage deep adversarial transfer learning approach. In the first step, the source model optimizes the network parameters by minimizing the source classification loss to generate high-quality pseudo-labels for each unlabeled auxiliary domain image. In the second step, the feature extractor is fixed, and the classifier and domain discriminator parameters are updated to maximize the inter-domain and intra-class variation loss. Fixing the classifier, updating the feature extractor by minimizing the difference between the two fixed classifiers in an attempt to generate features that are transferable with high discrimination.

The ability of deep learning networks to cope with dynamic changes in the sample space and unknown scenarios becomes a key measure of the level of intelligent diagnosis. Scene heterogeneity causes domain divergence in cross-domain sample distribution, resulting in poor model generalization performance when labeled data are used for cross-domain jamming scene perception. To address this issue, this section mainly introduces the details of our proposed network model. The cross-domain jamming generalization diagnosis model based on deep adversarial migration is shown in Figure 3, and the model is divided into a time-frequency image generation unit, a feature extraction module, a pseudo-label generation module, and a dual classifier adversarial verdict module.

In summary, we estimate the uncertainty weight of unlabeled samples based on the knowledge aggregation results of neighbour samples, reweight the classification loss and specify the direction of gradient optimization, giving high-quality pseudo-labeling to auxiliary domain jamming samples in heterogeneous scenarios. Upon comparing the classification outcomes, the focus of prediction attention is directed toward the classifier’s performance across various samples. The two classifiers execute gradient optimization in contrasting directions, contingent on their respective preferred classification clusters. Meanwhile, the adversarial metric factor is designed as the category difference measure to explicitly unify the two major tasks of improving diagnostic discriminate and reliability and to reduce the feature differences while ensuring that the content features are not contaminated. Meanwhile, the domain-level discriminate module implicitly pairs the edge probability distribution between its source domain and multiple auxiliary domains by designing the domain categorization loss and domain confusion loss.

### 3.1. Problem Definition

This paper proposes a transfer learning method for cross-domain jamming diagnosis, which can generalize the models trained on multiple source domains and auxiliary domains with different statistical characteristics to unknown scenarios. The research is based on the following assumptions. (1) The samples in the source domain are sufficient to establish a satisfactory diagnostic model. (2) The sample label set in the auxiliary domain and the target domain is a subset of all sample label sets in the source domain.

In LJCD-Net, obtain a fully labeled source domain $D_{LS}={\left\{\left(x_{LS}^{j},y_{LS}^{j}\right)\right\}}_{j=1}^{M}\left(j=1,2,\ldots ,M\right)$ and *N* unlabeled auxiliary domains ${\left\{D_{UAn}\right\}}_{n=1}^{N}={\left\{{\left\{x_{UAn}^{j}\right\}}_{j=1}^{M}\right\}}_{n=1}^{N}\left(n=1,2,\ldots ,N\right)$, $D_{UAn}$ denotes the *n*th unlabeled auxiliary domain, $x_{LS}^{j}$ and $y_{LS}^{j}$ are the *j*th sample and label in the source domain, respectively, $x_{UAn}^{j}$ denotes the *j*th sample in the auxiliary domain, and *M* is the number of samples contained in each domain. The auxiliary domain after pseudo-labeling is ${\left\{D_{PLAn}\right\}}_{n=1}^{N}={\left\{{\left\{x_{PLAn}^{j},y_{PLAn}^{j}\right\}}_{j=1}^{M}\right\}}_{n=1}^{N}$, and $y_{PLAn}^{j}$ denotes the *j*th sample pseudo-label after weighting in the auxiliary domain. Furthermore, denote $D_{T}={\left\{x_{i}^{T},y_{i}^{T}\right\}}_{i=1}^{N_{T}}\left(i=1,2,\ldots ,N_{T}\right)$ as the target domain with $N_{T}$ samples, which is inaccessible during training. For illustration, the sample space and label space of the source and auxiliary domains are uniformly denoted ${\left\{x_{j}^{S}\right\}}_{j=1}^{N}$ and ${\left\{Y_{j}^{S}\right\}}_{j=1}^{N}$, and $x^{T}$ and $y^{T}$ denote the sample space and label space of the target domain, respectively. Due to the domain divergence, the data space is made to drift, i.e., $x_{j}^{S}\neq x^{T}$. The aim of this study is to learn a mapping relation with generalization ability using $D_{LS}$ and ${\left\{D_{UAn}\right\}}_{n=1}^{N}$, which is capable of accomplishing the task of jamming diagnosis in unknown scenarios based on the existing data and labels.

### 3.2. Time-Frequency Image Generator Unit

The deep residual module, which incorporates convolutional layers, typically accepts a two-dimensional image as its input. Initially, we conducted a short-time Fourier transform (STFT) on the one-dimensional digital signals received at the receiver. This process converted these signals into a two-dimensional time-frequency spectral matrix, which was subsequently fed into the network for training and processing. The STFT splits the window of observed signals into small segments and assumes that the signals in each of the short segments are smooth.

Time-domain features elucidate the intensity and phase of a signal over time, illustrating the distribution of signal energy along the temporal axis. Conversely, frequency-domain features illuminate the frequency components, relative amplitude, minimum frequency, maximum frequency, and signal bandwidth of the signal. STFT employs a window function to depict the local time-frequency characteristics of the signal. The size of this window dictates the scale of the local features that are captured.

Taking any antenna branch signal y(n) as an example, the Hamming window [24] w(n) is chosen to segment the signal to obtain the signal $y_{F}\left(n\right)$ after the *F*th added window:

(9) $$y_{F}\left(n\right)=y\left(F\delta +n\right)w\left(n\right)$$

Among them, $n=0,1,\ldots ,w_{S}$, $w_{S}$ is the length of the Hamming window, and δ is the separation distance. The form of w(n) is as follows:

(10) $$w\left(n\right)=\left[k-\left(1-k\right)\cos\left(\frac{2\pi n}{\omega_{s}-1}\right)\right]R_{\omega_{w}}\left(n\right)$$

where $R_{\omega_{w}}\left(n\right)$ is a rectangular window of width $w_{S}$. Following the above steps, the signal overlap length $w_{s}-\delta$ between neighbouring windows, and the number of windows $N_{F}=\frac{L-\omega_{s}+\delta }{\delta }$. Thus, the original signal is segmented into $w_{s}-\delta$ segmented signals $y_{F}\left(n\right)$ of length $w_{s}$, $F=1,\ldots ,N_{F}$. Perform Fourier transform on the segmented local signal to obtain the windowed signal power spectrum:

(11) $$f_{F}\left(k\right)=\sum_{n=0}^{\omega_{S}-1}y_{F}\left(n\right)e^{-j2\pi kn/N},k=1,\ldots ,N/2-1$$

**Figure 4:**
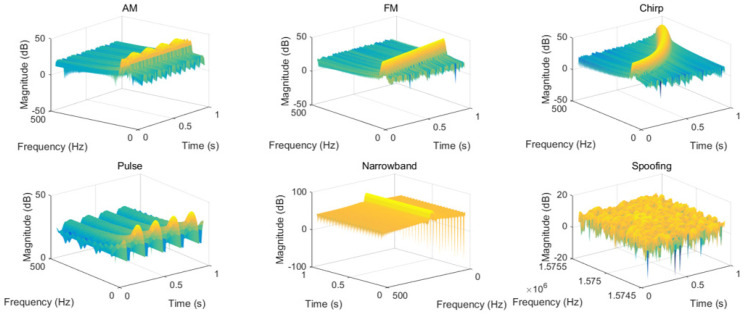
Time-frequency spectra of various types of jamming.

In the formula (N/2−1) is the number of points of Fourier transform. The longer the selected rectangular window, the higher the frequency resolution after transformation, but the lower the time resolution. The appropriate window length should be selected based on the time-frequency domain characteristics of the actual signal. Figure 4 shows the time-frequency spectrum of each type of interference.

$f_{F}\left(k\right)$ takes absolute values to obtain the linear spectrum S(k,F), which is further normalized to obtain G(k,F):

(12) $$G\left(k,F\right)=\frac{S(k,F)-\min(S)}{\max(S)-\min(S)}$$

The normalized linear spectrum yields a grayscale time-frequency map, and the color mapping yields an RGB time-frequency image. The size can be dynamically adjusted according to the network structure requirements in Section 3.3.

### 3.3. Feature Extraction Module

The feature extraction module is responsible for mapping the data to the feature space, and the spectrum matrix is sent to the feature extraction module as an equivalent two-dimensional image. The conventional structure of the convolutional neural network (CNN) is overly simplistic, rendering it insufficient for extracting stable jamming modal features from complex time-frequency images, we have segmented the feature extraction module into three distinct components: a two-dimensional CNN, a global attention module (GAM) [15], and a residual network.

Two-dimensional CNN is used to map low-dimensional jamming features in the time-frequency domain to high-dimensional space, and then the rectified linear unit (ReLU) nonlinear activation layer and downsampling pooling layer are introduced:

(13) $$H_{tf}=\mathrm{MaxPool}\left(\sigma \left(\Lambda \cdot X_{tf}+b\right)\right)$$

where $H_{tf}$ denotes the corresponding jamming feature in the time-frequency domain after downsampling, Λ is the trade-off weight, *b* is the bias that measures the fitting ability of the network, σ(·) is the Sigmoid function, maxpool(·) is the max-pooling layer operation, and $X_{tf}$ is the output of the time-frequency image generation module in Section 3.1.

**Figure 5:**
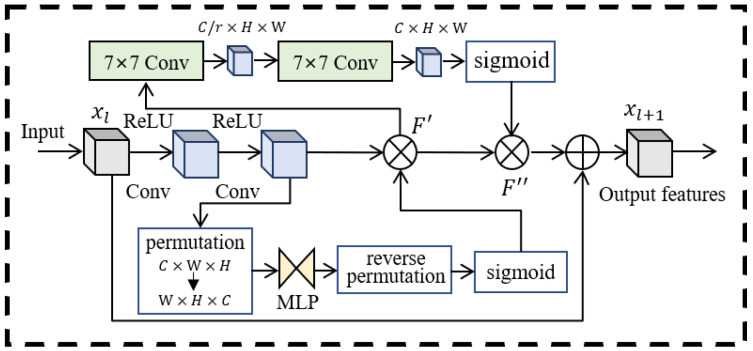
Global attention mechanism residual module.

GAM is introduced to perceive jamming information, as shown in Figure 5. The goal is to design an interactive feature that reduces information loss and amplifies the global dimension. The attention process is described as:

(14) $$F_{tf}=M_{s}\left(M_{c}\left(H_{tf}\right)⊗H_{tf}\right)⊗\left(M_{c}\left(H_{tf}\right)⊗H_{tf}\right)$$

$M_{s}$ and $M_{c}$ represent the spatial attention mechanism unit and channel attention mechanism unit, ⊗ denotes the element-by-element multiplication. The feature matrix derived from the time-frequency image via the convolutional network encompasses channels of varying significance. Consequently, the optimization of these features is achieved through the adjustment of weights to filter the feature channels.

(15) $$M_{c}\left(f\right)=\sigma \left(\mathrm{MLP}\left(\mathrm{AvgPool}\left(f\right)\right)+\mathrm{MLP}\left(\mathrm{MaxPool}\left(f\right)\right)\right)$$

*f* denotes the input feature vector and AvgPool denotes the average pooling layer. The vectors that represent features are derived through both the average and global maximum pooling, the spatial interdependence of cross-dimensional channels is enhanced using a two-layer multilayer perceptron (MLP).

Spatial attention is focused on identifying valuable image spaces, and we use two convolutional layers for spatial information fusion, each expressed as:

(16) $$M_{s}^{\prime }\left(f\right)=f^{7\times 7}\left(\left[\mathrm{AvgPool}\left(f\right);\mathrm{MaxPool}\left(f\right)\right]\right)$$

$M_{s}^{\prime }\left(f\right)$ is consistent with the spatial feature dimension, and the segmented feature vector $F^{\prime \prime }$ characterizes the importance of features in different regions of the image. The model training focuses on the global spatial features of the time-frequency map and the high-response channel by integrating the spatial channel attention mechanism. This effectively suppresses the occurrence of negative migration while ignoring the regions of the time-frequency map with lower mobility.

Enhancing the depth of the network enhances its diagnostic performance. The residual structure is employed to counteract the gradient degradation that occurs during the training phase. Furthermore, the relationship between the input and output feature vectors is also considered:

(17) $$F_{r-tf}=F\left(F_{tf},\left\{W_{i}\right\}\right)+F_{tf}$$

where $F_{r-tf}$ denotes the jamming features extracted from the residual structure. $F\left(\cdot ,\left\{W_{i}\right\}\right)$ is the residual mapping to be learnt.

### 3.4. Auxiliary Domain Label Weighting Module Based on Uncertainty Guidance

**Figure 6:**
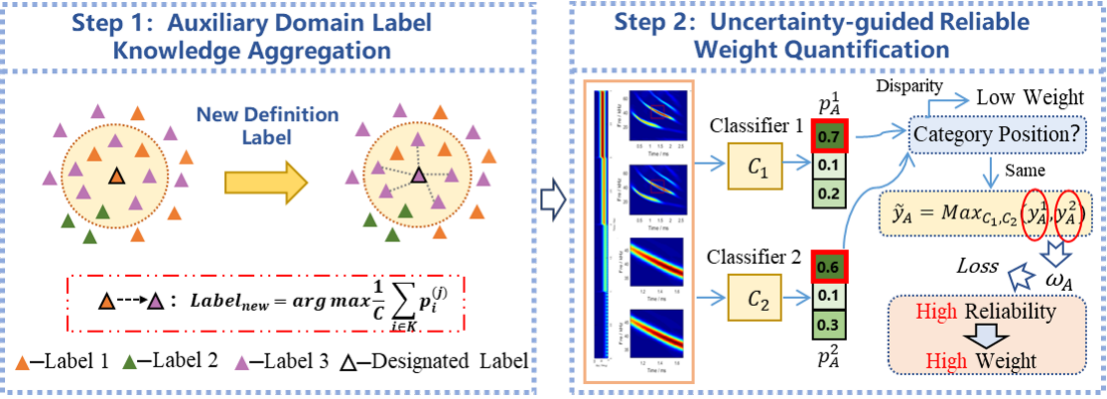
An auxiliary domain label weighting strategy based on uncertainty bootstrapping. In the first step, samples with adjacent features are more likely to have the same labels, and the refinement of pseudo-labels is completed by aggregating the knowledge of near-neighbour samples. In the second step, the gradient optimization direction of loss entropy is changed according to the infinite parameter of the probability vector, and the pseudo-labels with high confidence gain more trust.

Direct semi-supervised learning using hard pseudo-labels will lead to error propagation and convergence on the accuracy of the pseudo-labels. Figure 6 illustrates an uncertainty-guided high-quality pseudo-labeling weighting method (UGLW) for assisting domain samples in learning shared domain-invariant features in two steps.

One of the optimization objectives of UGLW is to minimize the source domain data state prediction error, defined as the cross-loss entropy:

(18) $$L_{A}^{C_{i}}=-\frac{1}{n_{l}}\sum_{i=1}^{n_{l}}\sum_{j=1}^{K}l\left\{y_{i}=j\right\}\log\left({\left[f\left(x_{i}|\theta \right)\right]}_{j}\right)$$

where $f\left(x_{i}|\theta \right)$ is the probability distribution predicted by unlabeled samples, ${\left[f\left(x_{i}|\theta \right)\right]}_{j}$ is the *j*th element of the probability vector, θ denotes the parameter on which the classifier can be trained, $n_{l}$ is the number of labeled samples, and l{·} is the judgment function, which returns 1 if the condition is true and 0 otherwise.

Pseudo-labeling is the process of assigning labels to unlabeled samples for further training. First use the source classification network to output a rough pseudo-label of the pseudo-labeled image sample:

(19) $$p_{i}=\underset{j\in L}{\mathrm{argmax}}{\left[f\left(x_{i}|\theta \right)\right]}_{j}$$

*L* is the number of categories, and the generated pseudo-labels are returned as labeled data to help model training. New markers are recomputed at the beginning of each epoch, and the loss function is defined as:

(20) $$L_{A}^{J_{P}}=-\frac{1}{n_{ul}}\sum_{i=n_{l}+1}^{n_{l}+n_{ul}}\sum_{j=1}^{K}l\left\{p_{i}=j\right\}\log\left({\left[f\left(x_{i}|\theta \right)\right]}_{j}\right)$$

$n_{ul}$ is the number of unlabeled samples.

The characteristics of semantically similar images should be situated within the adjacent feature space. This requirement is fulfilled by extracting the comparison vectors of analogous features from comparable samples. The knowledge aggregation method, which utilizes a soft-voting mechanism, is illustrated in step 1 of Figure 6. Specifically, given an unlabeled image $x_{a}$ and a weakly enhanced image $t_{tf}$ randomly drawn from the distribution $T_{tf}$, the vector $z_{n}=f_{a}\left(t_{tf}\left(x_{a}\right)\right)$ is obtained after feature extraction of the weakly enhanced image $t_{tf}\left(x_{a}\right)$. Library *K* contains a set of randomly selected feature and prediction vector pairs ${\left\{z_{i}^{\prime },p_{i}^{\prime }\right\}}_{i=1}^{M}$. To maintain the stability of the feature information, the slowly transforming momentum model $g_{a}^{\prime }\left(\cdot \right)=h_{a}^{\prime }\left(f_{a}^{\prime }\left(\cdot \right)\right)$ is used to update the storage in the library. The cosine distance between the features of $x_{a}$ and the features in the library Neighbour is then calculated to confirm the update.

(21) $$D_{cos}=1-\frac{A\cdot B}{∥A∥∥B∥}=1-\frac{\sum_{j=1}^{n}A_{j}\times B_{j}}{\sqrt{\sum_{j=1}^{n}{\left(A_{j}\right)}^{2}}\times \sqrt{\sum_{j=1}^{n}{\left(B_{j}\right)}^{2}}}$$

In the comparison framework, *A* and *B* represent the two feature vectors. The *M* samples exhibiting the smallest distance are chosen as neighbours. The pseudo-labels of $x_{a}$ are then optimized by leveraging the knowledge derived from these neighbouring samples:

(22) $${\hat{p}}_{a}^{\left(m\right)}=\frac{1}{C}\sum_{i\in J}p_{i}^{\left(m\right)}$$

J denotes the set of neighbours for the chosen augmented samples, while *m* signifies the averaging operation applied to each class. To enhance the quality of pseudo-tags, markers with the highest number of classes are identified $y_{A}=\mathrm{argmax}{\hat{p}}_{a}^{\left(m\right)}$.

Given the fact that high-quality pseudo-labels are resistant to changes during training iterations, two classifiers, $C_{1}$ and $C_{2}$, are designed. These classifiers share the same structure but differ in their initial parameters. Both $C_{1}$ and $C_{2}$ are trained concurrently under identical conditions to yield two sets of predictions, denoted as $\left\{{\hat{p}}_{i}^{a1}\right\}$ and $\left\{{\hat{p}}_{i}^{a2}\right\}$. The consistency of pseudo-labels across the same sample is predominantly observed in both sets of collections. Samples exhibiting disparate pseudo-labels are deemed to have been erroneously predicted at least once and, consequently, they are excluded from this backpropagation process, denoted as:

(23) $$I_{i}=\left\{\begin{array}{c} 1,\mathrm{if}\left({\hat{p}}_{i}^{a1};\theta_{1}\right)\cap \left({\hat{p}}_{i}^{a2};\theta_{2}\right)\neq ⌀\\ 0,\mathrm{otherwise} \end{array}\right.$$

where $\theta_{1}$ and $\theta_{2}$ denote the parameters of $C_{1}$ and $C_{2}$, respectively, and $\theta_{1}\neq \theta_{2}$. As shown in step 2 in Figure 6, the samples participate in the training only when ${ℶ}_{i}=1$.

To further improve the predictive ability of the model, the reliability weights of the unsupervised samples are quantified by estimating their uncertainty, where high-confidence pseudo-labeled samples are assigned more trust as a direction of gradient optimization, defining the weights of each pseudo-labeled sample $\left(x_{a},y_{A}\right)$ as

(24) $$\omega_{i}={\left[1+\frac{L\left({\tilde{y}}_{A}^{i}-1\right)}{L-1}\right]}^{1+\left(L-1\right)\frac{\mathrm{epoch}}{\mathrm{epochs}}}$$

where epoch is the current training round, and epochs are the pre-determined total rounds, which together form the scale factor (epoch/epochs) that adaptively adjusts the thresholds as the training progresses, accelerating the gradient optimization path toward high-quality labeling, and ${\tilde{y}}_{A}^{i}$ denotes the probability that the pseudo-labeled sample $x_{a}$ belongs to the target category:

(25) $${\tilde{y}}_{A}^{i}={\mathrm{Max}}_{C_{1},C_{2}}\left({∥{\hat{p}}_{i}^{a1}∥}_{\infty },{∥{\hat{p}}_{i}^{a2}∥}_{\infty }\right)$$

The weights obtained are reassigned to the loss function, thereby modulating the influence of pseudo-labeled samples on the training content for each calendar element. This is probabilistically guided by the classification loss.

(26) $$L_{A}^{J_{P}}=-\frac{1}{n_{ul}}\sum_{i=n_{l}+1}^{n_{l}+n_{ul}}\omega_{i}\sum_{j=1}^{K}l\left\{p_{i}=j\right\}\log\left({\left[f\left(x_{i}|\theta \right)\right]}_{j}\right)=-\frac{1}{n_{ul}}\sum_{i=n_{l}+1}^{n_{l}+n_{ul}}\omega_{i}\times \sum_{j=1}^{K}l\left\{p_{i}=j\right\}\log\left({\left[f\left(x_{i}|\theta \right)\right]}_{j}\right)$$

As a result, low-quality labels receive low weights in backpropagation, and high-quality samples will be given larger weights.

The model leverages high-quality pseudo-labels in a timely manner during the training phase. To circumvent the a posterior collapse problem, we follow the state-of-the-art strategy of optimizing the subsequent regular terms:

(27) $$L_{A}^{div}=E_{x_{a}\in X_{a}}\sum_{i=1}^{n_{l}+n_{ul}}{\tilde{p}}_{q}\log{\tilde{p}}_{q},{\tilde{p}}_{q}=E_{x_{a}\in X_{a}}\sigma \left(f_{a}\left(t_{tf}\left(x_{a}\right)\right)\right)$$

The overall loss function used for weighted training of LJCD-Net pseudo-labeled samples is.

(28) $$L_{UGLW}=L_{A}^{C_{i}}+\alpha_{P}L_{A}^{J_{P}}+\alpha_{div}L_{A}^{div}$$

where $\alpha_{P}$ and $\alpha_{\mathrm{div}}$ represent the trade-off parameters that can be further optimized. The pseudo-labels generated are utilized to facilitate the learning of inter-domain knowledge, taking into account the preferences of clusters as delineated in Section 3.5.

In particular, in semi-supervised learning, the number of fully labeled samples is much less than the number of unlabeled samples, resulting in the unsupervised term dominating the loss function and the model paying more attention to samples with pseudo-labeling. In order to overcome this problem, in the same batch of training, the numbers of source domain and auxiliary domain samples are set to fixed numbers $n_{1}$ and $n_{2}$, respectively, and the model is updated through multiple iterations to meet the supervision term in the loss function, ensuring supervised learning robustness of the process.

### 3.5. Adversarial Learning of Dual Classifiers Based on Implicit Distributional Alignment

After obtaining high-quality pseudo-labels for the auxiliary domain samples, LJCD-Net designs a domain discriminator and category adversarial implicit alignment module, where the two adaptive units learn shared domain-invariant knowledge by aligning the joint distributions of features and categories across the domains, which consists of a feature generator *G* and a domain discriminator *D* with the same structure of the dual classifiers $M_{1}$ and $M_{2}$.

The existing two-classifier adversarial learning paradigm first trains the feature extractor and two classifiers with the same structure using labeled samples, then fixes the feature extractor *G*, trains the two classifiers, and employs the $L_{1}$ paradigm [25] to measure the divergence between the two domains to maximize the output gap between the target data on the classification predictions. Finally, the classifiers are fixed and the feature extractor parameters are optimized to minimize the output difference between the target data on the two classifiers.

The above method is theoretically closer to the HΔH divergence, but since the two classifiers only consider the differences between the classifiers in the mini competition game, the similarity of the output does not guarantee the accuracy of the target sample, even if the difference between classifiers is small, and the target sample may be matched to the wrong category. Therefore, based on the pseudo-labels generated in Section 3.4, the prediction attention is shifted to the degree of excellence of the classifier on different samples. According to the optimal set of classification clusters, the two classifiers complete opposite optimizations in their respective fields.

In each update, given a batch of auxiliary domain samples ${\left\{x_{i}^{a}\right\}}_{i=1}^{C^{a}}$, the predicted labels obtained from the two classifiers are ${\left\{p_{i,1}^{a}\right\}}_{i=1}^{C^{a}}$ and ${\left\{p_{i,2}^{a}\right\}}_{i=1}^{C^{a}}$, respectively. Due to the lack of real labeling information in the auxiliary domain, the current labeling information is approximated using high-quality pseudo-labels described in Section 3.4. Kullback–Leibler (KL) divergence [26] is used to measure the similarity between each sample prediction vector and the pseudo-label vector, $KL\left(P|Q\right)=\sum p_{i}\log\left(p_{i}/q_{i}\right)$. If the KL divergence is large, it means that the prediction result is far away from the pseudo-label. For a given sample, a smaller KL scatter indicates better play of the classifier. Therefore, the samples are preferred into two clusters based on the performance of the dual classifier:

(29) $$\begin{array}{cc} & C_{1}^{a}=\left\{x_{i}^{a}|KL\left({\tilde{y}}_{i}^{a},p_{i,1}^{a}\right)<KL\left({\tilde{y}}_{i}^{t},p_{i,2}^{a}\right)\right\}\\ & C_{2}^{a}=\left\{x_{i}^{a}|KL\left({\tilde{y}}_{i}^{a},p_{i,1}^{a}\right)>KL\left({\tilde{y}}_{i}^{t},p_{i,2}^{a}\right)\right\} \end{array}$$

$C_{1}^{a}$ and $C_{2}^{a}$ represent the set of samples with better classification performance on $M_{1}$ and $M_{2}$, respectively. We attempt to further reduce the fuzzy samples by maximizing the prediction difference within both clusters. Taking the update of classifier $M_{1}$ as an example, it outperforms $M_{2}$ on Cluster A and therefore further maximizes the prediction difference with $M_{2}$, whereas $M_{2}$ performs worse on Cluster A and needs to further reduce the prediction difference with $M_{1}$. Classification loss of $M_{1}$

(30) $$\min_{M_{1}}L_{\mathrm{cre}-1}\left(D_{t}\right)=-L_{\mathrm{dis}}\left(C_{1}^{a}\right)+L_{\mathrm{dis}}\left(C_{2}^{a}\right)$$

$\left\{-L_{\mathrm{dis}}\left(C_{1}^{a}\right)\right\}$ moves the prediction of $M_{1}$ on Cluster A away from $M_{2}$, while $\left\{L_{\mathrm{dis}}\cdot \left(C_{2}^{a}\right)\right\}$ moves the prediction of $M_{1}$ on Cluster B closer to $M_{2}$, in addition:

(31) $$L_{cre}\left(X\right)=E_{x\in X}\mathrm{disc}\left(p_{1},p_{2}\right)$$

$\mathrm{disc}\left(p_{1},p_{2}\right)$ is the difference in predictions of the bi-classifier, typically measured by the $L_{1}$ paradigm or the sliding Wasserstein distance [27]. Similarly, the classification loss of $M_{2}$

(32) $$\min_{M_{2}}L_{\mathrm{cre}2}\left(D_{t}\right)=L_{\mathrm{dis}}\left(C_{1}^{a}\right)-L_{\mathrm{dis}}\left(C_{2}^{a}\right)$$

Parameter initialization consists of a fully connected layer activated by the ReLU function, and training is performed using the cross-entropy function of fully labeled data:

(33) $$L_{T}^{M}=-\frac{1}{n_{s}}\sum_{i=1}^{n_{s}}y_{i}^{s}\log\left(M_{k}\left(G\left(x_{i}^{s}\right)\right)\right)$$

In summary, the loss of our bi-classifier clustering adversarial strategy is:

(34) $$\begin{array}{cc} & \min_{M_{1}}E_{x_{i}^{s}\in D_{s}}L_{T}^{M}\left(M_{1}\left(G\left(x_{i}^{s}\right)\right),y_{i}^{s}\right)+L_{\mathrm{cre}1}\left(D_{t}\right)\\ & \min_{M_{2}}E_{x_{i}^{s}\in D_{s}}L_{T}^{M}\left(M_{2}\left(G\left(x_{i}^{s}\right)\right),y_{i}^{s}\right)+L_{\mathrm{cre}2}\left(D_{t}\right) \end{array}$$

The above scheme achieve as reliable diagnostic accuracy, but the classifier’s prediction confidence for source domain samples is much higher than that for auxiliary domain samples. Taking the three-classification situation as an example, the prediction result of [0.85, 0.1, 0.05] is likely to occur in $D_{LS}$, while the classification situation of [0.4, 0.3, 0.3] is more likely to occur in $D_{UAn}$. To address this problem, we design an adversarial metric factor based on the classification prediction results:

(35) $$\begin{array}{c} L_{T}^{Adv}=E\left[\frac{{∥P_{1}-P_{2}∥}_{2}}{{∥P_{k}∥}_{\infty }}\right]\\ P_{1}={\mathrm{Softmax}}_{x_{i}^{a}∼D_{UAn}}\left(M_{1}\left(G\left(x_{i}^{a}\right)\right)\right)\\ P_{2}={\mathrm{Softmax}}_{x_{i}^{a}∼D_{UAn}}\left(M_{2}\left(G\left(x_{i}^{a}\right)\right)\right) \end{array}$$

where $P_{k}$ is the classification result on the better performing classifier for that sample, using the above calculation as a measure of inter-class difference. Specifically, the introduction of adversarial metric factors as category difference measures can clearly unify the two tasks of improving diagnostic discriminability and credibility, reducing category differences while ensuring that content features are not contaminated.

The domain discrimination module implicitly aligns the edge probability distribution between the source and auxiliary domains by designing the domain classification loss and domain confusion loss. Classification loss of domain discriminator *D*:

(36) $$L_{do}=-\left(\frac{1}{n_{s}}\sum_{i=1}^{n_{s}}\log\left(1-D\left(G\left(x_{i}^{s}\right)\right)\right)+\frac{1}{n_{a}}\sum_{j=1}^{n_{a}}\log\left(D\left(G\left(x_{j}^{A}\right)\right)\right)\right)$$

where $n_{s}$ is the number of source domain samples and $n_{a}$ is the number of auxiliary domain samples. The domain confusion loss is formulated by calculating the cross-entropy between pseudo-labels and predicted distributions to maximize the confusion of the boundaries between different domains:

(37) $$C_{do}=\lambda \left(L_{\mathrm{domain}}-\frac{1}{n_{s}}\sum_{i=1}^{n_{s}}\log\left(D\left(G\left(x_{i}^{s}\right)\right)\right)-\frac{1}{n_{a}}\sum_{j=1}^{n_{a}}\log\left(1-D\left(G\left(x_{j}^{A}\right)\right)\right)\right)$$

where λ is the metric parameter. By optimizing the respective loss functions of the domain discrimination and category adversarial implicit alignment modules, the joint alignment of conditional and marginal probability distributions is achieved:

(38) $$\min_{G,D}L_{T}^{D}=\min_{D}L_{do}+\min_{G}C_{do}$$

Prior studies [28] have demonstrated that feature vectors characterized by the highest singular values predominantly influence the transferability of feature representations. Conversely, those with lower singular values tend to be excessively penalized during adversarial training, resulting in a diminished level of distinguishability. The BSP scheme is employed to attenuate the feature vectors associated with the highest singular values, thereby facilitating a relative enhancement of the remaining vectors:

(39) $$L_{T}^{bsp}=\sum_{n=1}^{N}\sum_{q=1}^{Q}{\left(\sigma_{q}^{n}\right)}^{2}$$

where $\sigma_{q}^{n}$ denotes the *q*th largest singular value. The difference compared to the original intention of using BSP in [28] is that we apply it for the first time to domain generalization problems and exploit its potential in the jamming diagnosis.

Taken together, the final optimization objective of this subsection is:

(40) $$J=L_{T1}^{cre}-L_{T2}^{cre}+\gamma L_{T}^{Adv}-\lambda L_{T}^{D}+\xi L_{T}^{bsp}$$

where γ, λ and ξ are adjustable weights. Let $\theta_{G}$, $\theta_{M}$ and $\theta_{D}$ be the parameters of the feature extractor, classifier, and domain discriminator, respectively, the network optimization problem is formulated as:

(41) $$J\left(\theta_{G}^{\prime },\theta_{M}^{\prime },\theta_{D}^{\prime }\right)=\min_{\theta_{G},\theta_{M},\theta_{D}}\left(L_{T}^{cre}\left(\theta_{G},\theta_{M}\right)+\gamma L_{T}^{Adv}\left(\theta_{G},\theta_{M}\right)\right.\left.-\lambda L_{T}^{D}\left(\theta_{G},\theta_{D}\right)+\xi L_{T}^{bsp}\left(\theta_{G}\right)\right)$$

The gradient descent method is employed to update the model parameters in each batch. This approach facilitates the joint optimization of several losses, including the source-auxiliary domain classification loss, class adversarial loss, domain discrimination loss, and maximum singular value regularization loss.

During the offline training phase, jamming signal data from various domains are converted into time-frequency 2D matrices. Subsequently, the training process is halted by iterating through the set of training parameters until the loss function J exhibits a variation of less than 0.1%. In the online classification phase, these time-frequency data are inputted into the trained LJCD-Net to predict the type of jamming.

## 4. Generation of Training and Test Data

**Figure 7:**
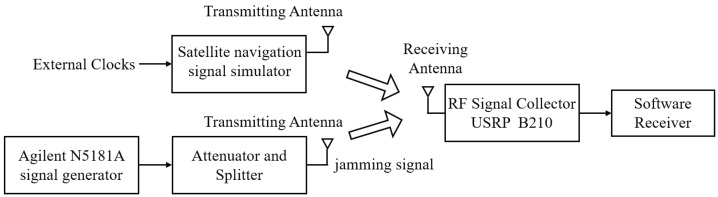
Generation of SOI and jamming data using hardware test benches.

In order to evaluate the performance of the algorithm in this article, a hardware test bench was used to generate training and testing datasets, as shown in Figure 7. An Agilent N5181A (Agilent Technologies, Santa Clara, CA, USA) signal generator was used to generate jamming, and a tunable attenuator was used to control the generated signal power. The receiving antenna collects the GNSS or 5G positioning signals generated by the satellite navigation simulator and feeds them to the RF signal collector USRP B210 (National Instruments, Austin, TX, USA) for processing. The software receiver is a device designed by the author’s research group for SOI research. The sampling frequency is no higher than 50 MHZ, the front-end bandwidth is between 10 and 20 MHz, the ADC resolution is 12 bits, and the data incoming from the USRP are recorded in real time.

In a typical positioning receiver, four signals are typically required to complete the position solution. The number of signals available to the receiver at varying complexity levels is represented by from 1 to 4 hybrid SOIs. Pseudo-codes of the same PRN sequence are chosen to generate spoofed signals but with different time delays and Doppler.

To facilitate the SNR calculation, all channel coefficients are normalized and generated by a land mobile channel object designed based on the ITU-R Recommended Format P.681-11 [29]. In the context of the relative movement between the source and receiver, a semi-Markov chain is employed, which allows for the generation of signals that alternate between high and low quality. High-quality signals have direct paths within line of sight, while low-quality signals are characterized by severe obstruction or complete non-line-of-sight. Taking BeiDou B1 as an example, the parameters of the generated signal are set as follows: the carrier frequency point is selected as 1575.42 MHz, and the channel sampling rate is 1.023 MHz. Multipath signals that add reflection and refraction have an impact on the received SOI. The channel fading does not obey the statistical characteristics, and the channel state undergoes unknown distortion. The multipath is assigned a random number, ranging from 1 to 5, the delays of adjacent paths are randomly distributed over a pseudocode length.

The channel coefficients for both jamming and spoofing incorporate Rayleigh and Rician fading samples, which generate six distinct types of jamming: AM, FM, chirp, narrowband, pulse, and spoofing. These jammings are generated in a randomly distributed manner within a predefined parameter space. To simulate random delays in spoofed jamming navigation messages, positioning messages are generated with a duration of 1 bit and the first 1 ms of the signal is taken for training. The delay in the navigation message was determined by a stream of bits in integer multiples of the duration, with each bit multiplied by a random number of plus or minus 1. For each signal type, 8000 samples for the training set and 1000 samples for the test set are generated. Table 1 lists the parameter space definitions for each jamming, where U(a,b) denotes a uniform distribution within [a,b].

## 5. Experimental Results and Analysis

### 5.1. Lack of Available Source Domains

To validate the generality of the model proposed in this paper, before investigating the diagnostic capability of the generalized model, we consider a special case where the training dataset contains only a fully labeled source domain and the test set samples are still unknown, where the sufficient prerequisites of domain generalization and domain adaptation for migration are not met. Accordingly, the training set samples cover five in-band squelch-type jammings, AM, FM, chirp, NB, and pulse, which are trained under the conditions of a signal-to-noise ratio (SNR) of 0 dB and a jamming-to-signal ratio (JSR) of 5 dB, and the test set samples obey the JSR∼U(−15,10)dB. In this study, we selected several jamming identification methods for comparative analysis to elucidate the diagnostic superiority of LJCD-Net in scenarios devoid of auxiliary domain samples. For the sake of impartiality and rationality, all network model-based methods employed identical parameter structures. The specific configurations of these comparison techniques are detailed below.

M1: Joint fractional order Fourier transform and trap for jamming detection (FR-TRAP) scheme [6];
M2: Classical decision tree (DT) scheme based on thresholding [30];
M3: Time-domain constructed residual network suppression based jamming identification (TD-RNN) scheme [10];
M4: Deep subdomain adaptive network (DSAN) [11];
M5: DANN [31].

The proposed method and the comparison method are implemented in the PyTorch framework. During the network training process, the Kaiming initializer is used to preset network parameters. The parameter update uses an SGD optimizer with a momentum of 0.9 and a weight decay of $5\times 10^{-4}$. The first-stage hyperparameters $\alpha_{P}$ and $\alpha_{div}$ are both empirically set to 1, and the hyperparameters γ, λ, and ξ are empirically set to 1, 0.1, and $1\times 10^{-5}$, respectively. The epoch and batch sizes of all methods were set to 100 and 64, respectively, with an initial learning rate of $1\times 10^{-3}$.

The methodology proposed in this study necessitates the use of multiple classes of unlabeled auxiliary samples during the generalization phase to facilitate the acquisition of domain-invariant knowledge. In instances where only a single fully labeled domain is incorporated, the training process is confined solely to the source domain, and pseudo-label generation does not incorporate migration training. We conducted 1000 Monte Carlo simulations to assess the recognition performance of various algorithms under varying JNRs. The accuracy was determined as the average of the results from five classes of suppressed jamming diagnosis.

**Figure 8:**
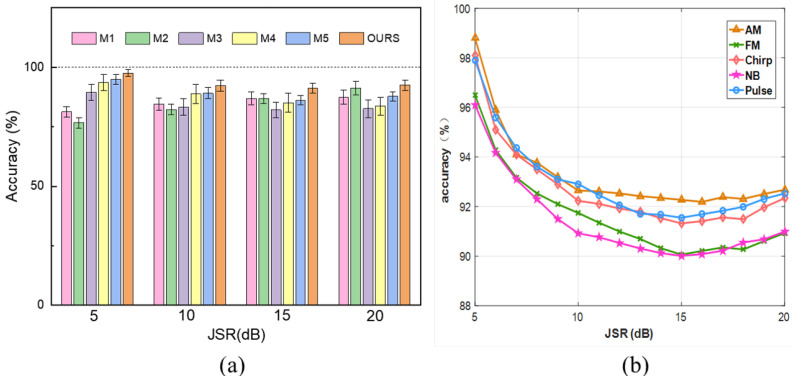
Diagnostic ability of the methods for jamming in the case of only one fully labeled source domain: (**a**) Recognition accuracy of various methods under different JSR. (**b**) Recognition accuracy of LJCD-Net in dealing with various types of jamming.

The recognition accuracies of from M1 to M5 and the methods in this paper at different JSRs are shown in Figure 8a. It can be seen that M1 and M2, as representatives of the traditional jamming diagnosis methods, have different performances compared to M3, M4, and M5 in the face of the increase in JSR from 5 dB to 20 dB. Traditional jamming diagnosis methods are not influenced by the number of source domains or cross-domain tests. In contrast, FR-TRAP estimates the instantaneous frequency and recovers the phase information of jamming through time-frequency plane rotation. Meanwhile, DT classifies the jamming step by comparing characteristic parameters with a priori thresholds until each category set contains only one type of jamming. As JSR escalates, the time-frequency ridges of the jamming signal become progressively more pronounced and the number of eigenvalues expands. Consequently, the diagnostic performance of these aforementioned methods incrementally improves.

TD-RNN is not affected by the number of source domains, but still because the training and testing scenarios do not comply with independent and identical distribution, the jamming diagnosis performance of JSR from 10 to 20 dB is about 7% lower than that of JSR at 5 dB. DSAN and DANN, as typical migration learning networks, outperform M1 to M4 in diagnostic performance at JSR = 5 dB, but reduce the recognition accuracy by more than 9% in other test scenarios due to the lack of the number of source domains and domain divergence between training and test scenarios, and do not improve with JSR. LJCD-Net is also limited by the number of source domains and test scenarios, but it adds a global attention mechanism residual module to extract more stable jamming modal features, and obtains a better diagnostic performance when JSR = 5 dB, in unknown scenarios The test is also inferior to traditional and transfer learning methods.

Figure 8b demonstrates the recognition accuracy of the network model proposed in this paper under different JSR when dealing with various types of jamming. Since the model is only trained at JSR = 5 dB, the neural network does not perform well in the face of unfamiliar probability density distributions and the diagnostic performance decreases as the difference between the marginal probability and conditional probability distributions increases, despite the fact that the increase in jamming power leads to a more pronounced feature representation. In this particular scenario, the diagnostic performance of the conventional method when JSR reaches 20 dB is similar to that of our method.

### 5.2. Sufficient Number of Available Source Domains

With a sufficient number of available source domains, we only need one fully labeled source domain and multiple unlabeled auxiliary domains to complete the generalization. In order to more intuitively demonstrate the advantages of LJCD-Net in unknown environments, two sets of migration experiments are designed: migration between different spoofing jamming types and migration between different JSR datasets under the same jamming type. Different methods with similar network architecture and experimental settings to the proposed method were selected for comparison, and ablation experiments were conducted to verify the effectiveness of each module. All models use the same network architecture and hyperparameters as LJCD-Net, and the methods are shown in Table 2.

In the first part, from N1 to N5 are designed as comparative experiments based on the existing domain adaptation or the domain generalization extension to demonstrate the effectiveness and superiority of LJCD-Net. It is worth mentioning that N1 will have different results depending on the selected source domain. Choose the best one among these results for comparison. N2 uses the same feature extraction and classification module as the scheme in this paper. N3, as an upgrade of DANN, constrains the inter-domain distribution distances in an adversarial manner. N4 extracts domain-invariant and discriminative features from multiple source domains with potential differences and shows great potential in jamming recognition. N5 adds an attention mechanism and a residual network to DANN, and it has been shown to have excellent performance in jamming recognition. In the second part, we conduct the ablation experiments from A1 to A4 to verify the necessity of the key strategies in LJCD-Net, keeping the parameter settings of the remaining modules.

#### 5.2.1. Migration Experiments between Different Spoofing Jamming Types

Malicious spoofing scenarios are fully considered and spoofed signals are generated using the same PRNs with different time delays, Doppler frequencies, and multipaths. We set the six main jamming types of jamming-free single SOI (single SOI), jamming-free multiple SOI, Doppler shift, time delay, joint Doppler-time shift, and multipath as the target identification objects for LJCD-Net and the control method.

**Figure 9:**
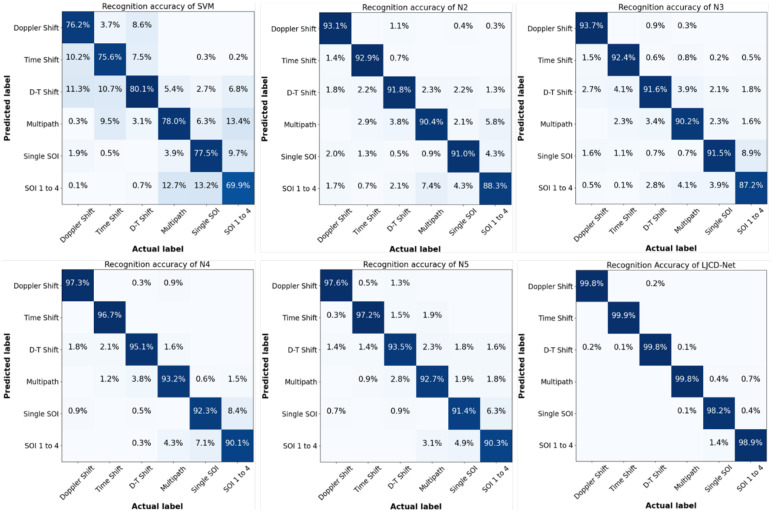
Comparison of classification accuracies of SVM (**top left**), from N2 to N5 (**in descending order**) and LJCD-Net (**bottom right**) for six signal or jamming types, namely, single SOI, SOI from 1 to 4, Doppler offset, time delay, combined Doppler-time offset, and multipath.

The classification obfuscation diagrams for the six algorithms are illustrated in Figure 9, with support vector machines (SVMs) being included in the comparison as a prevalent method of classification. This is due to the fact that both N1 and N2 employ convolutional neural networks (CNNs) as independent extraction networks, performing analogous functions in the diagnostic process. The accurate prediction accuracies and their respective incorrect prediction percentages for each spoofing category are prominently displayed in Figure 9. It is evident from this figure that the diagnostic accuracy of the proposed LJCD-Net surpasses that of the other five methods when the training and testing data fall within the same distribution. LJCD-Net demonstrates an average recognition rate of 99.4% across all signal patterns, while the recognition accuracies for alternative methods vary between 76.2% and 94.1%. N5, a prevalent method for spoofing jamming recognition, yields commendable results in diagnosing Doppler and time delay. However, its performance is subpar when compared to N4 in scenarios involving multipath-oriented and multiplexed signal overlapping spoofing. N2 and N3 employ distinct CNN architectures as the foundational network for feature extraction and recognition, while they exhibit comparable recognition accuracies across various spoofing types, a singular convolutional architecture proves inadequate for extracting stable features and making accurate judgments in the context of intricate time-frequency images. Notably, the average diagnostic accuracy is approximately 89% when dealing with multipath and multiplexed SOI mashups. Support vector machines (SVM) are frequently employed to detect suppression jamming, demonstrating commendable performance in non-line-of-sight scenarios and multipath clustering, but with an average identification accuracy of 76.2% in the face of subtly differentiated spoofing jamming.

A/B/C/D/E denotes single SOI, Doppler offset, time delay, Doppler-time offset, and multipath spoofing, and tasks from T1 to T10 denote **A**/B/C/D→E, **B**/A/C/D→E, **A**/B/C/E→D, **A**/B/C→D, **A**/B/D/E→C, **A**/B/E→C, **A**/C/D/E→B, **A**/C/E→B, **B**/C/D/E→A, and **B**/C/D→A, respectively. The results in Table 3 show that the average diagnostic accuracy (96.40%) of the proposed scheme (LJCD-Net) in this paper is significantly better than that of other methods, and the diagnostic efficacy has been improved by 8.74%, 7.67%, and 2.71%, respectively, when compared with N1, N3, and N5. Furthermore, the LJCD-Net exhibits superior stability compared to other comparative methods across a majority of tasks. This suggests that the model is proficient in generating uncontaminated shared knowledge and demonstrates a greater resilience to unfamiliar diagnostic scenarios.

(1) Both N1 and N2 perform the cross-domain diagnosis task with a traditional CNN architecture, and the overall performance is far from what we expect. N1 performs worse in task **A**/C/E→B, which is because the model can only learn delay features from, and has a weak generalization to, jamming features in the frequency offset category, and the huge specificity leads to poor diagnosis results. The overall robustness of N2 is better than that of N1 (3.04% improvement), which is due to the multi-domain adversarial training that improves the robustness of the model. Although N1 exhibits a superior diagnostic performance compared to N2 (a 5.05% improvement), this may be attributed to the fact that merely amalgamating source domains with significant distributional disparities into a singular training domain could potentially undermine the network’s generalization capabilities.
(2) The utilization of adversarial training, as opposed to conventional domain metrics, can significantly enhance the capacity of feature extractors to assimilate shared knowledge. This is evidenced by the comparative analysis between LJCD-Net and N3. The DANN-based method incorporates a discriminator compared to CNN, and the results show that N4 obtains better diagnostic results (4.5% improvement) compared to N3 in most of the tasks, suggesting that the parameter adaptation of the network model is effective in improving the generalization of the model. N5 further incorporates the attention mechanism and residual learning into DANN, thereby reducing the standard error. This improvement in diagnostic performance over N4 is notable. However, it proves ineffective in tasks **A**/C/E→B and **B**/C/D→A. This suggests that while N5 possesses superior general knowledge portability and broad applicability, it also poses challenges in learning specific feature representations that are more beneficial for target domain diagnosis.
(3) The difficulty in generalizing **A**/B/E→C and **A**/C/E→B may be due to the large span of jamming features in the migration task, and the time-frequency features learned from the source and auxiliary domains are still deviating from or failing to satisfy the diagnostic needs of the target domain. The more obvious the inter-domain divergence is, the more difficult the migration becomes.
(4) The only difference between **A**/B/C/D→E and **B**/A/C/D→E is the different labeling domains, and the poorer migration effect of the latter is due to the fact that the Doppler shift cannot obtain more general time-frequency features compared to the single SOI, and cannot generate more reliable pseudo-labels to be used in the next stage of confrontation. Overall, the use of single SOI as the labeling domain can help us obtain satisfactory clustering results.

**Figure 10:**
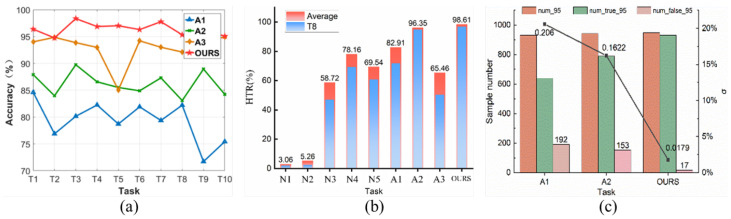
Overall analyses in migration experiments with different spoofing types. (**a**) Ablation experiments. (**b**) Feature quality analysis. (**c**) Misclassification analysis.

Figure 10a and Table 3 corroborate the efficacy of each strategy and module. The absence of precise and dependable auxiliary domain pseudo-labels in A1 leads to a marked decrease in the diagnostic outcomes. This could be attributed to the weighting strategy, which is grounded on uncertainty guidance, effectively mitigating misclassifications of the training samples and serving as a guiding mechanism for the accurate classification of target samples. A2 and A3 remove the clustering confrontation and confidence measure loss, respectively, resulting in a rather ambiguous prediction of the sample by the model, and the lack of either one prevents a trade-off between accuracy and reliability.

A good feature should identify its own category with a high confidence probability. Due to the lack of criteria for judging feature quality, reference [23] identifies features with a prediction confidence of 95% or higher as high-quality features. We use the proportion of high-quality features (HTR) in correctly classified samples to judge the overall quality of model-generated features:

(42) $$\mathrm{HTR}=\frac{{\mathrm{num}}_{\mathrm{true}\text{-}95}}{{\mathrm{num}}_{\mathrm{true}}}$$

${\mathrm{num}}_{\mathrm{true}-95}$ is the number of samples that are classified correctly with a confidence level of 95% or more. Figure 10b illustrates the percentage of high-quality features in the correctly classified samples. Due to the unavailability of valid classification boundaries, it is difficult for N1 and N2 to produce high-quality predictions. A3 removes the loss of adversarial metrics. T8, for example, shows a significant decrease in the prediction confidence when the source domain diverges from the target domain, proving that $L_{T}^{Adv}$ is a core module for improving the diagnostic confidence. The mean HTR of LJCD-Net across various tasks is 98.16%, suggesting that the semantic features are the least affected, thereby ensuring highly dependable cross-domain diagnosis.

To further validate the discriminatory nature of the model, the number of samples with a prediction confidence of more than 95% but incorrectly diagnosed was analysed. The misclassification rate σ:

(43) $$\sigma =\frac{{\mathrm{num}}_{\mathrm{false}\text{-}95}}{{\mathrm{num}}_{95}}$$

Taking T8 as an example, Figure 10c demonstrates the misclassification of A1 and A2 with our scheme. Compared with A1 and A2, LJCD-Net reduces the misclassification probability by 18.81% vs. 14.33%, respectively, which further illustrates that our pseudo-label weighting strategy with dual-classifier clustered adversarial approach plays a key role in improving the discriminative nature of diagnosis.

#### 5.2.2. Migration Experiments between Different JSR Datasets with the Same Jamming Type

Time-varying power increases the dynamics and uncertainty of jamming features, subverts the inherent time-frequency knowledge learnt by the DNN model in the training samples, and greatly affects the performance of various types of jamming and SOI diagnosis. Considering the case where both suppression and spoofing jammings exist, six jammings, AM, FM, chirp, NB, pulse, and spoofing (Doppler-time) are diagnosed and then averaged, and the cross-domain diagnostic task settings are shown in Table 4.

**Figure 11:**
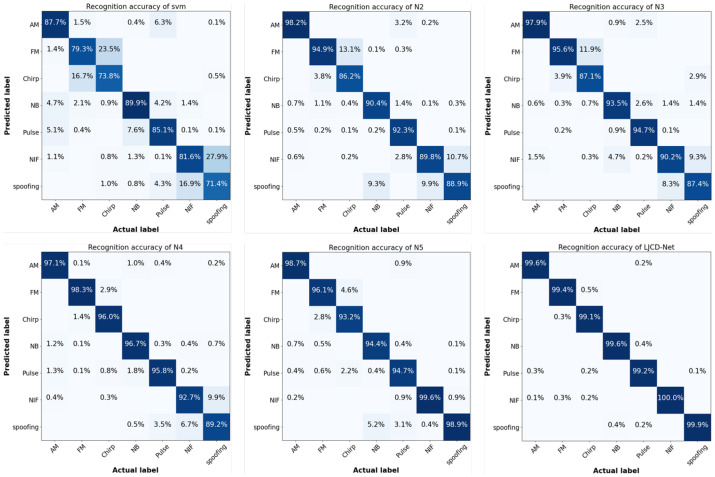
Comparison of classification accuracies of SVM (**top left**), from N2 to N5 (**in descending order**), and LJCD-Net (**bottom right**) for seven signals or jammings, AM, FM, chirp, NB, pulse, NIF, and spoofing.

The diagnostic performance of SVM, N2 to N5, and LJCD-Net for each type of jamming is illustrated in Figure 11. NIF stands for a SOI without jamming, and NIF before spreading is shown as thermal noise on the time-frequency diagram due to the fact that the power of suppressed jamming is much higher than that of the useful signal. It can be seen that the average diagnosis success rate of LJCD-Net for all types of jamming is 99.5%, which is better than 95.1% of DT-DDG and 96.5% of Attention-RDANN. Among them, the chirp signal is often mistaken for an FM signal. The possible reason is that our TF window period is 1 ms and the chirp duration defined in Table 1 is 5 ms. This mismatch makes the jamming time-frequency characteristic representation with a longer scanning period closer for FM signals. Secondly, the pulse signal is susceptible to the misclassification of AM, NB, and spoofing due to the fixed-pulse bandwidth of 1 MHz and the narrowest pulse peak period ranging from approximately 1 to 10 μs. This often leads to the assumption that these signals share similar time-frequency characteristics with the AM signal. When the power of the spoofing jamming is high, it affects the correct diagnosis of pulse in short cycles as well, and its own classification is often spoofed by NIF. The SVM is unsatisfactory as an jamming diagnostic tool, especially for the two easily confused decisions of FM and chirp, NIF and spoofing. The average recognition accuracy is only between 76.6% and 76.5%. The preceding analysis has revealed that factors contributing to misclassification encompass discrepancies in feature representation, which arise from varying TF window selections, and temporal-frequency ambiguity, resulting from power fluctuations. In practical applications, the diagnostic accuracy can be further stabilized through the normalized setting of various jamming target parameters.

Table 5 demonstrates that the average diagnostic accuracy (96.94%) of LJCD-Net is significantly better than other methods in each task. Comparing T11 and T13, it is not difficult to find that suppressing the jamming by generalizing from high to low JSR targets results in a better diagnosis, and the reason for the lower overall recognition performance of T12 may be that it is difficult to share the knowledge of targets at 110 dB with the source domain samples of JSR from 40 to 60 dB. Upon comparing T14, T15, and T16, it becomes evident that augmenting the number of training sources does not invariably enhance the diagnostic performance. Instead, it impedes the acquisition of universal diagnostic knowledge due to an inability to effectively address the distributional disparities among them. N4 proficiently supports the acquisition of generic diagnostic knowledge by evaluating the transferability of multiple sources in the context of “distant sources”. However, N4 applicability diminishes when the target sample is deemed not to exhibit a greater similarity to the “distant source”. It is inferred against T17 to T20 that when the source and auxiliary domain sample knowledge is similar, the model focuses on the learning specific features of both, ignoring the potential differences that may exist in other domains, which need to be addressed by introducing diversity and variability. A4 removes the global attention residual module and performs ablation experiments on the feature extraction module proposed in Section 3.3. The results show that the traditional CNN has a single structure, which cannot distinguish between the channel as well as the importance of spatial information, and is insufficient to extract stable subtle features in the face of generalized scenarios with a large gap between the source-target domains, such as T12 and T16. T17 to T20 show that the CNN is unable to pay attention to the regions of higher mobility in the time-frequency map in the face of the spoofing jamming with lower power, which even leading to the occurrence of gradient degradation.

**Figure 12:**
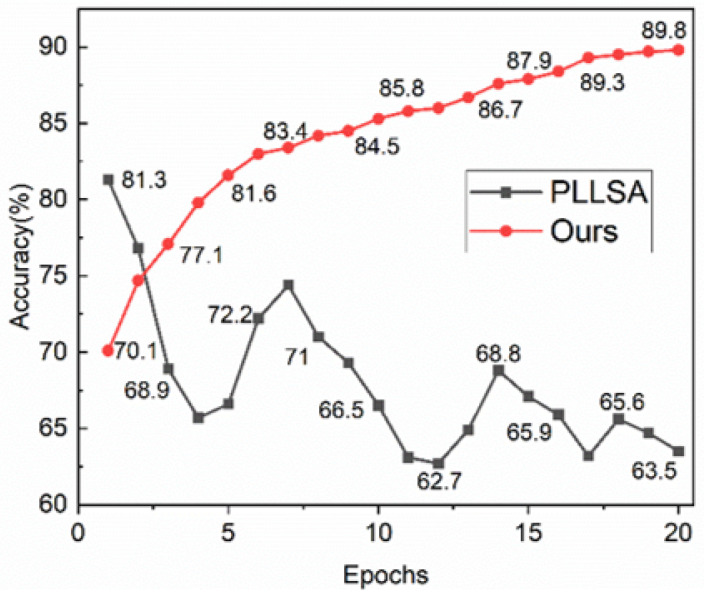
Refinement of pseudo-labeling accuracy. Noise is not dealt with in PLLSA [34], where model overfitting generates erroneous pseudo-labels. Instead, we guide the gradient direction of the pseudo-label update optimization with the help of neighbourhood knowledge, which mitigates the effect of noisy samples and thus gradually improves the accuracy and confidence of the pseudo-labels.

Figure 12 plots the accuracy trend of the optimized pseudo-labels during the iterative process. PLLSA [34] is highly affected by pseudo-label noise, which leads to difficulty in convergence and gradual degradation of performance during the iterative process. Contrarily, UGLW directs the gradient direction of the pseudo-label update optimization utilizing neighbourhood knowledge. This approach substantiates its efficacy in enhancing noise robustness. Thus, our scheme is able to endow the auxiliary domain samples with high-quality pseudo-labeling, which, in turn, guides the next generalization task.

**Figure 13:**
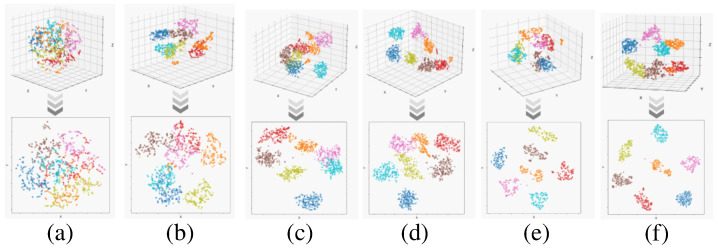
Feature visualisation of T13 test results. (**a**) N1. (**b**) N2. (**c**) N3. (**d**) N4. (**e**) N5. (**f**) LJCD-Net.

The feature visualisation results of the proposed method and the comparison method are shown in Figure 13, with the first row showing the 3D visualisation results, which depicts the degree of inter-domain separation and intra-class aggregation more clearly compared to the 2D visualisation. Different colours represent different categories of interference. As shown in Figure 13a,b, the use of a single source or simple merging of information from all domain samples results in the apparent confusion between the different categories, when the knowledge learnt from the source and auxiliary domains does not work effectively in the target domain. On the other hand, as shown in Figure 13f, the proposed scheme in this paper shows a significant improvement in inter-class separation and intra-class aggregation compared to other methods, which indicates that the shared high-level feature representations of the same class of disturbances across different domains are effectively mined, and the target samples can be benefited from the generic diagnostic knowledge, which leads to an improved diagnostic performance.

**Figure 14:**
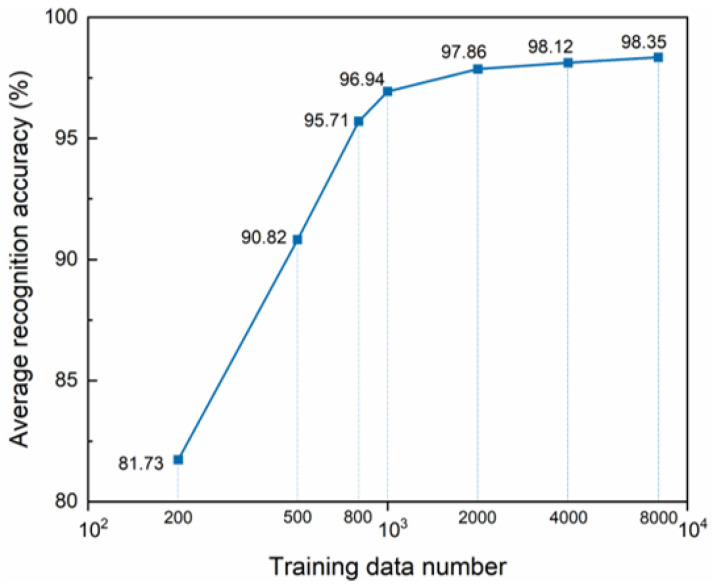
Average correct recognition rate versus amount of training data.

The efficacy of the training model is evaluated using datasets of varying sample sizes, with the final average recognition success rate illustrated in Figure 14. The data clearly demonstrate that as the volume of training data escalates, so too does the average recognition success rate. This observation aligns with the prevailing principle that a larger number of samples for the learning model correlates positively with an enhanced recognition accuracy. From a practical application point of view, training with more samples leads to a longer training time with a greater workload, and we expect to accomplish the projected goal with less data. Figure 14 shows that when the number of samples of each type is 800, the average recognition success rate is around 95.7%. After the number of samples exceeds 2000, the slope of the increase in recognition success rate is very slow as the training samples increase significantly. Considering that the auxiliary domain samples do not need to be labeled, this amount of data is not difficult to prepare and train in terms of duration in practical applications. Table 6 demonstrates the average recognition accuracy of T16 with different amounts of training data. As the number of samples increases further, the recognition accuracy increases slowly while the standard deviation decreases significantly and converges gradually.

## 6. Conclusions

In this study, we seek to provide a generalized jamming diagnosis method for cross-domain heterogeneous scenarios to support jamming diagnosis in real-life positioning scenarios using jamming information data collected in the laboratory. The proposed method aims to use a fully labeled source domain and multiple unlabeled auxiliary domains to generate a shared feature representation with a generalization ability, and tries to solve the problems of the low quality of pseudo-labels, and the poor discriminative and low confidence of classification models. The experimental results show that LJCD-Net can effectively identify suppression and spoofing jamming in the face of unknown scenarios, and outperforms the existing classical and advanced schemes.

To our knowledge, this is the first work of adversarial domain generalized network in jamming diagnosis. In the future, we will start from the joint high-confidence PNT spatial and temporal service of GNSS and 5G to provide a higher accuracy, availability, and reliability for the new spatial and temporal network. However, there is still a need to provide more experimental data and test scenarios for the generalized perception model, and how to overcome the negative migration phenomenon as well as further mining of jamming features to guide the diagnosis performance will be the next research direction.

## Figures and Tables

**Table 1:** Definition of the jamming parameter space.

Jamming Type	Parameter Setting
AM	$f_{p_{k}}∼U\left(0.1,1\right)\;\mathrm{MHz}$
	Two sets of parameters are normalized
FM	$\beta_{k}∼U\left(0.1,0.9\right)$
	$f_{m_{k}}∼U\left(0.1,1\right)\;\mathrm{MHz}$
Chirp	$f_{\min}=1\;\mathrm{MHz}$
	$f_{\max}=25\;\mathrm{MHz}$
	$T_{swp}∼U\left(0.05,5\right)\;\mathrm{ms}$
	a=±1
NB	$\Omega \left(f\right)=\left\{\begin{array}{cc} \mu , & \left|f-f_{0}\right|<B/2\\ 0, & \mathrm{otherwise} \end{array}\right.$
	B=4 MHz
	$z_{nb}\left[n\right]∼N\left(0,0\right)$
Pulse	B=1 MHz
	$\alpha_{k}∼U\left(0.8,1.2\right)$
	a=U(0.02,1) μs
	$M_{p}=2$
Spoofing	$\psi_{k}∼U\left(-\infty \forall^{\prime },\infty \forall^{\prime }\right)$
	$f_{kp}∼U\left(-\infty \Delta^{\prime },\infty \Delta^{\prime }\right)$

**Table 2:** Comparison method.

Method	Description
N1	CNN training by a single source domain data
N2	Train CNN by merging data from multiple domains together
N3	CNN + MMD [32] trained by generalization from multiple domain data
N4	DT-DDG [22], combining dynamic weighting strategies and batch spectral penalty regularization terms for adversarial training
N5	Attention-RDANN [33], adding attention mechanisms with residual networks
A1	Remove uncertainty-guided weighting strategies
A2	Remove bi-classifier clustering adversarial strategies
A3	Remove of adversarial metric loss $L_{T}^{Adv}$
A4	Remove global attention residual module

**Table 3:** Diagnostic accuracy of each method under different migration tasks (%).

Task	N1	N2	N3	N4	N5	A1	A2	A3	LJCD-Net
**T1**	90.72 ± 3.91	89.32 ± 0.81	91.71 ± 1.03	94.87 ± 2.85	95.58 ± 0.45	84.59 ± 7.06	87.89 ± 3.26	94.01 ± 3.11	**96.37 ± 0.92**
**T2**	87.05 ± 4.57	86.45 ± 1.73	88.20 ± 1.67	90.31 ± 2.26	91.91 ± 1.82	76.87 ± 10.86	83.96 ± 2.91	**94.85 ± 3.99**	94.74 ± 1.73
**T3**	92.09 ± 4.11	91.99 ± 1.87	92.26 ± 1.99	95.03 ± 3.94	**98.70 ± 1.38**	80.14 ± 6.99	89.74 ± 3.11	93.86 ± 4.07	98.34 ± 0.52
**T4**	88.25 ± 5.92	89.76 ± 2.82	88.57 ± 3.71	92.87 ± 3.55	95.12 ± 2.71	82.24 ± 9.64	86.56 ± 2.67	92.97 ± 2.78	**96.86 ± 1.14**
**T5**	90.41 ± 4.96	91.07 ± 1.45	91.39 ± 2.36	95.46 ± 2.57	**97.18 ± 2.60**	78.70 ± 8.79	85.50 ± 3.62	85.03 ± 3.66	97.01 ± 0.95
**T6**	83.06 ± 6.05	84.33 ± 2.24	85.20 ± 2.97	93.18 ± 2.11	90.16 ± 3.42	81.90 ± 9.37	84.89 ± 4.06	94.20 ± 2.92	**96.29 ± 0.59**
**T7**	91.31 ± 3.98	86.36 ± 1.88	91.67 ± 1.18	94.92 ± 2.89	96.43 ± 1.95	79.36 ± 7.56	87.29 ± 3.61	93.00 ± 2.57	**97.76 ± 0.34**
**T8**	68.49 ± 5.70	79.91 ± 3.22	82.67 ± 3.45	91.47 ± 3.05	86.66 ± 4.00	82.21 ± 7.55	83.07 ± 4.68	92.11 ± 3.82	**95.32 ± 1.31**
**T9**	88.38 ± 5.02	86.12 ± 1.06	90.83 ± 1.50	92.52 ± 2.57	95.45 ± 1.89	71.70 ± 9.02	88.93 ± 4.14	93.90 ± 3.41	**96.21 ± 0.98**
**T10**	82.79 ± 5.34	80.04 ± 2.16	84.81 ± 2.64	91.67 ± 2.77	89.71 ± 3.23	75.42 ± 7.63	84.22 ± 2.02	94.95 ± 3.24	**95.07 ± 1.13**
Average	86.26 ± 4.96	86.54 ± 1.92	88.73 ± 2.25	93.23 ± 2.86	93.69 ± 2.34	79.31 ± 8.45	86.21 ± 3.41	92.89 ± 3.26	**96.40 ± 1.00**

**Table 4:** Cross-domain diagnostic tasks between different JSR datasets.

Jamming Type	Task	Source (dB)	Auxiliary (dB)	Target (dB)
suppressive	T11	40	50/60	70
	T12	40	50/60	110
	T13	70	50/60	40
	T14	40	50/70/110	60
	T15	40	50/70	60
	T16	40	50/70/110	100
spoofing	T17	5	10/20	15
	T18	5	8/10	20
	T19	10	11/12	5
	T20	8	10/15	5

**Table 5:** Diagnostic accuracy of methods in the migration task (%).

Task	N1	N2	N3	N4	N5	A4	LJCD-Net
T11	79.36 ± 3.89	84.51 ± 2.04	91.73 ± 1.96	92.82 ± 1.51	96.20 ± 1.06	95.34 ± 1.75	**98.43 ± 0.61 **
T12	77.03 ± 4.46	81.72 ± 3.16	88.65 ± 2.58	92.64 ± 0.93	**94.81 ± 1.89**	89.41 ± 2.38	94.53 ± 1.50
T13	80.22 ± 4.23	85.68 ± 1.75	90.83 ± 2.71	92.56 ± 2.42	97.86 ± 1.21	96.10 ± 0.96	**99.02 ± 0.14**
T14	84.20 ± 5.15	76.72 ± 2.88	86.04 ± 2.31	**95.12 ± 1.87**	92.43 ± 2.75	91.86 ± 1.22	94.62 ± 0.36
T15	80.77 ± 4.75	86.83 ± 2.96	91.46 ± 3.56	93.55 ± 2.80	96.31 ± 0.99	96.05 ± 1.89	**98.52 ± 1.17**
T16	85.94 ± 4.69	80.28 ± 3.23	86.92 ± 3.41	76.04 ± 4.60	92.56 ± 1.89	88.63 ± 1.91	**95.79 ± 0.91**
T17	83.47 ± 4.97	87.31 ± 3.24	93.27 ± 2.37	94.90 ± 1.01	96.80 ± 1.95	92.15 ± 1.56	**98.87 ± 0.16**
T18	74.62 ± 5.11	79.06 ± 3.86	85.62 ± 3.38	93.55 ± 2.25	95.40 ± 1.03	91.83 ± 1.79	**96.92 ± 1.00**
T19	79.86 ± 6.25	76.63 ± 4.72	84.41 ± 3.34	90.02 ± 3.65	87.14 ± 3.98	87.46 ± 3.35	**95.20 ± 2.67**
T20	83.99 ± 4.70	83.71 ± 2.02	89.08 ± 2.66	94.10 ± 2.78	95.26 ± 1.95	91.72 ± 0.86	**97.45 ± 0.32**
Average	80.95 ± 4.82	82.25 ± 2.99	88.80 ± 2.83	91.53 ± 2.38	94.48 ± 1.87	92.06 ± 1.77	**96.94 ± 0.88**

**Table 6:** Average recognition accuracy of T16 with different amounts of training data (%).

Training Data Number	2000	5000	8000	12,000	20,000
performance	93.92 ± 1.93	94.81 ± 1.20	95.79 ± 0.91	96.03 ± 0.35	96.29 ± 0.26

## Data Availability

The original contributions presented in the study are included in the article, further inquiries can be directed to the corresponding authors.
